# Reference values for spirometry in elderly individuals: a cross-sectional study of different reference equations

**DOI:** 10.1186/s40248-017-0112-5

**Published:** 2018-01-09

**Authors:** Joana Belo, Teresa Palmeiro, Iolanda Caires, Ana L. Papoila, Marta Alves, Pedro Carreiro-Martins, Maria A. Botelho, Nuno Neuparth

**Affiliations:** 1 Lisbon School of Health Technology, Polytechnic Institute of Lisbon, Lisbon, Portugal; 2 Chronic Diseases Research Centre, CEDOC, NOVA Medical School/Faculdade de Ciências Médicas, Universidade Nova de Lisboa, Lisbon, Portugal; 3 Epidemiology and Statistics Analysis Unit, Research Centre, Centro Hospitalar de Lisboa Central, EPE, Lisbon, Portugal; 4 CEAUL, NOVA Medical School/Faculdade de Ciências Médicas, Universidade Nova de Lisboa, Lisbon, Portugal; 5 Immunoallergoly Department, Dona Estefânia Hospital, Centro Hospitalar de Lisboa Central, EPE, Lisbon, Portugal

**Keywords:** Elderly, Spirometry, Reference eqs., GLI, NHANES III, ECSC

## Abstract

**Background:**

Spirometry is the single most important test for the evaluation of respiratory function. The results are interpreted by comparing measured data with predicted values previously obtained from a reference population. Reference equations for spirometry have been discussed previously. The aim of this study was to compare reference values based on National Health and Nutrition Assessment Survey (NHANES III), European Community of Steel and Coal (ECSC), and Global Lung Initiative (GLI) equations in an elderly sample population.

**Methods:**

Subjects from the Geriatric Study on Health Effects of Air Quality in elder care centres who met the inclusion criteria were enrolled. Spirometry was performed according to international guidelines. The forced vital capacity, forced expiratory volume in 1 s, and FEV_1_/FVC ratio were reported as percentages of the predicted value, and the lower limit of normality was calculated.

**Results:**

Out of 260 elderly patients, 69.6% were women; the mean age was 83.0 ± 6.46 years with an age range of 65–95 years. The lowest %FVC and %FEV_1_ values were obtained using the GLI reference equations. However, when NHANES III equations were used, the FEV_1_/FVC ratio was higher than ratios obtained from GLI and ECSC equations. The prevalence of airway obstruction was highest using ECSC equations, while GLI equations demonstrated more restrictive defects.

**Conclusions:**

The present study showed meaningful differences in the reference values, and consequently, in the results obtained using NHANES III, ECSC, and GLI reference equations. The spirometry interpretation was also influenced by the reference equations used.

## Background

Spirometry is the single most important test for the evaluation of respiratory function and screening of general respiratory health [1]. Spirometry interpretation compares measured data with previously obtained predicted values, preferably obtained from a reference population [2]. The predicted values, not only for spirometry but also for other lung function tests, vary with age, sex, standing height, and ethnic group, and are obtained using reference equations [2]. The selection of adequate reference equations for spirometry has been discussed previously [3] and was updated in 2012 with the publication of all-age multi-ethnic reference equations for spirometry (the Global Lung Initiative [GLI]) [4]. These equations included a larger population, more ethnicities, and ages ranging from 3 to 95 years. Swanney and Miller [5], in their editorial for the *European Respiratory Journal*, emphasized the statistical approach of the GLI, which included a larger age range, as young and elderly subjects show greater variability in predicted values than middle-aged subjects. Therefore, the result was the publication of reference equations for a wide age range that attempted to rectify the relative lack of reference data for elderly subjects [6, 7]. Before the GLI publication, specific studies of elderly populations showed substantial variability in predicted values, which may lead to interpretation inaccuracies [7–10] that become pertinent in elderly populations as they continue to be studied. The adoption of new GLI equations and the possible effect on lung function test interpretation had already been studied in adults aged less than 85 years [11, 12], children, and adolescents [13]. To our knowledge, there are no significant data available regarding the effects of spirometry interpretation in an extremely aged population.

The reference equations should be derived from a population with the same characteristics as the population of the tested individuals; at least 53 studies on this subject were published between 1995 and 2004 [14]. In most lung function laboratories (LFLs), the choice of reference values varies; recommendations from the National Health and Nutrition Assessment Survey (NHANES III) [15] are commonly used in the United States of America (USA), and those from the European Community of Steel and Coal (ECSC) [16] are commonly used in Europe. The population ages and ethnic characteristics are the primary differences between the GLI, NHANES III, and ECSC data. The NHANES III equations are derived from a population sample of Caucasian, Mexican, and African-American descent between 18 and 80 years of age, while the ECSC equations are derived from a Caucasian sample between 18 and 70 years of age. The GLI was a task force endorsed by five international societies and used a multi-ethnic sample from 3 to 95 years of age.

As each recommendation used a different population, it is clear that the reference equations obtained must also be different, creating potential problems in result interpretation for laboratories, clinicians, and technologists. Thus, it is important to identify the implications of adopting different equations. According to Brazzale et al. [17], changing to the GLI equations could affect the interpretation of spirometry values, as their direction and magnitude are dependent on which reference data are used in practice.

The aim of this study was to compare spirometry results expressed as percentages of the predicted values and spirometry interpretation using ECSC, NHANES III, and GLI reference equations in elderly subjects.

## Methods

### Study design and setting

This cross-sectional study is part of the Geriatric Study in Portugal on Health Effects of Air Quality (GERIA) that was conducted in Portugal in two phases. In Phase I, 33 elder care centres (ECCs) from Lisbon and 20 from Porto were selected using proportional stratified random sampling (by parish) from the 151 ECCs included in the Portuguese Social Charter. In Phase II, a cluster analysis of the 33 ECCs from Lisbon included in Phase I was performed to select 18 ECCs. This study reports the results from Phase II, which included the spirometry studies.

The GERIA project was approved by the Ethics Committee of NOVA Medical School/Nova University. The procedures followed were in accordance with those of the Code of Ethics of the World Medical Association (Declaration of Helsinki). The database was registered with the Portuguese Data Protection Authority (CNPD). Elderly subjects and their caregivers were informed of the study’s purpose, procedures, and risks, and informed consent was obtained.

### Participants

The spirometry tests were performed between November 2013 and March 2014. Elderly subjects who were residents of ECCs for more than 6 months and aged between 65 and 95 years were included. This upper limit was defined in consideration of the GLI’s age range of 3–95 years. Predicted values from NHANES III and ECSC data were extrapolated beyond the ages of 80 and 70 years, respectively. According to American Thoracic Society (ATS) and European Respiratory Society (ERS) recommendations [2], elderly subjects who had any of the following conditions were excluded: 1. a myocardial infarction in the preceding month, 2. chest or abdominal pain due to any cause, 3. oral or facial pain exacerbated by a mouthpiece, 4. stress incontinence, or 5. dementia or a confused state. Subjects were also excluded if they had an unstable cardiovascular and respiratory status; recent thoracic, abdominal, or eye surgery; a recent pulmonary embolism; thoracic, abdominal, or cerebral aneurysms; disorders that would affect test performance (such as haemoptysis of unknown origin, nausea, or vomiting); a resting pulse rate ≤ 60 bpm or ≥100 bpm; a pulse oximetry value ≤90%; a systolic and diastolic blood pressure ≥ 160/100 mmHg; or an absence of the cognitive capacity necessary to understand the procedure.

### Data sources

The examinations were performed by qualified and certified technicians with sufficient training to ensure that proper testing procedures were followed. The technicians understood common signs of pulmonary disease identification and acquired pulmonary function data management. Height was measured according to ATS/ERS recommendations [2]. For subjects with thoracic cage deformities and those who used a wheelchair, the arm span was measured and the arm span to height ratio (1.06 for men and 1.03 for women) was applied to the estimate the height. The spirometry results were measured using ATS/ERS standardized procedures and quality control [1, 18] with a Vitalograph®Compact (Vitalograph, Buckingham, United Kingdom).

### Variables

The forced vital capacity (FVC), forced expiratory volume in 1 s (FEV_1_), and FEV_1_/FVC ratio were reported after being validated by a committee of experts composed of medical doctors and health technicians. The percentage of the predicted value for %FVC and %FEV_1_ and the corresponding lower limit of normality (LLN) are presented. The published regression equations from ECSC, NHANES III, and GLI were used to calculate the predicted values. The LLN was calculated for use with the ECSC reference values by using the following equation: predicted – (1.64 × standard deviation [SD]) [16] and by using the predicted values obtained from each equation. The published regression equations from NHANES III provided the predicted values and the LLN [15]. GLI software was used to calculate predicted values and the LLN (http://www.ers-education.org/guidelines/global-lung-function-initiative/tools/excel-individual-calculator.aspx).

Airway obstruction was identified when the FEV_1_/FVC < LLN for each reference equation. In clinical medicine, “normal” is defined as the range of values that includes 95% of a healthy population. Therefore, a LLN indicating a reduced FVC, FEV_1_, and FEV_1_/FVC is defined as a result below the fifth percentile of the predicted value [16]. A reduced vital capacity does not confirm the presence of a restrictive pulmonary defect, but a normal or increased FEV_1_/FVC with a reduced FVC may suggest lung restriction [14]. In this study, a spirometric restrictive pattern was considered when the FVC ≤ LLN and the FEV_1_/FVC ≥ LLN.

### Statistical methods

Categorical data are presented as frequencies (percentages) and continuous variables are presented as the mean ± SD. A Bland-Altman plot was analysed to evaluate the agreement between the spirometry results. Because of the presence of proportional bias (when the slope of the regression of the differences of the averages is not zero) and heteroscedasticity (when the scatter of values for differences increases progressively as the average values increase), Bland-Altman plots were constructed using the ratios of the values from each of two methods plotted against their averages. Obstruction and spirometric restrictive pattern agreement were calculated from the kappa statistic using the following interpretation criteria: 0–0.2: slight; 0.21–0.40: fair; 0.41–0.60: moderate; 0.61–0.80: good; and 0.81–0.99: very good. An α = 0.05 was considered significant. The statistical analysis was performed using SPSS Statistics for Windows, version 22.0 (IBM Corp., Armonk, NY, USA) and MedCalc for Windows, version 15.0 (MedCalc Software, Ostend, Belgium).

## Results

Out of the 817 elderly subjects invited to participate in spirometry testing, only 307 accepted. However, 45 elderly subjects were excluded because they did not produce a quality spirometry result. Moreover, as the predicted values obtained for Caucasians are consistently different from those obtained for non-Caucasians [19, 20], two elderly subjects who were not Caucasian were also excluded from the analysis. A total of 260 elderly subjects were included in this study. Subjects who underwent spirometry had a mean age of 83.0 ± 6.46 years, and 181 (69.6%) were women.

### Spirometry results as a percentage of the predicted value

By observing spirometry results as a percentage of the predicted value for FVC, FEV_1_, and FEV_1_/FVC (%FVC, %FEV_1_, and %FEV_1_/FVC) across the different reference equations stratified by sex, it is possible to conclude that the GLI provided the lowest values for %FVC and the ECSC provided the highest values, with a difference of 18.0%. This difference increased to 23.4% among the women. The %FEV_1_ results obtained using the ECSC equation followed the same tendency, with increases of 16.2% and 20.2% in all subjects and in female subjects, respectively. When observing the %FEV_1_/FVC results, those derived from NHANES III had the highest means and those from ECSC had the lowest means. To clarify, the greatest difference was observed in women and the %FEV_1_/FVC mean from the ECSC equation was 16.3% lower than that from NHANES III (Table 1).

**Table 1:** Spirometry results expressed as percentage of predicted values

		NHANESIII	ECSC93	GLI12
	n	Mean (SD)	Mean (SD)	Mean (SD)
Total				
FVC (%)	260	87.5 (26.7)	98.4 (30.5)	80.4 (22.9)
FEV_1_ (%)	260	87.5 (31.5)	92.0 (33.5)	75.8 (25.2)
FEV_1_/FVC (%)	260	97.7 (14.3)	85.9 (13.9)	92.3 (13.5)
Female				
FVC (%)	181	89.5 (26.9)	104.6 (30.5)	81.2 (22.4)
FEV_1_ (%)	181	89.4 (31.6)	97.4 (34.2)	77.2 (24.9)
FEV_1_/FVC (%)	181	98.48 (13.3)	82.2 (11.2)	93.1 (12.5)
Male				
FVC (%)	79	82.5 (25.6)	84.2 (25.3)	78.5 (23.9)
FEV_1_ (%)	79	83.3 (30.9)	79.6 (28.4)	72.5 (25.8)
FEV_1_/FVC (%)	79	96.0 (16.2)	94.3 (15.9)	90.6 (15.3)

**Fig. 1:**
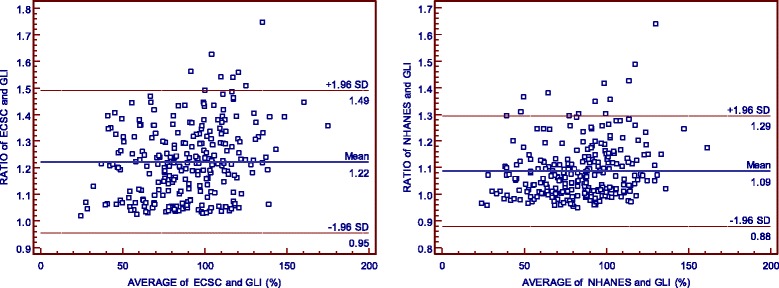
Agreement between the results expressed as percentage of predicted values for FVC obtained by GLI12 and ECSC93 equations (on the left), and by GLI12 and NHANESIII equations (on the right), using Bland-Altman plots.

**Fig. 2:**
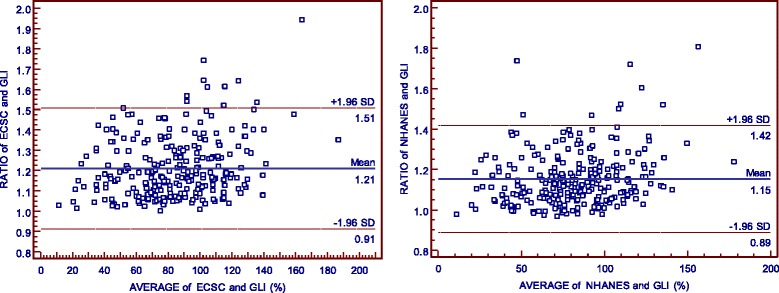
Agreement between the results expressed as percentage of predicted value for FEV_1_ obtained by GLI12 and ECSC93 equations (on the left), and by GLI12 and NHANESIII equations (on the right), using Bland-Altman plots

**Fig. 3:**
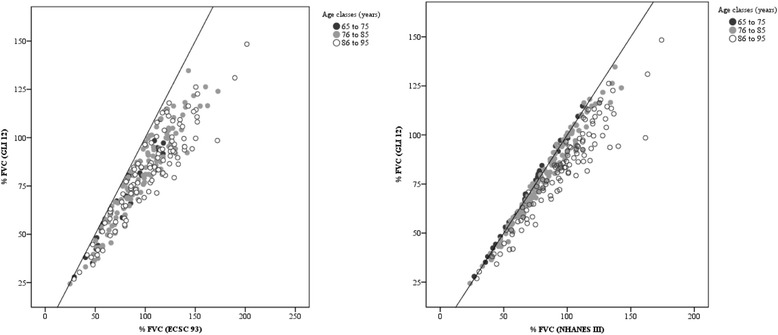
Scatter plot associating the results as a percentage of the predicted value for FVC obtained by GLI12 reference equations with those obtained by ECSC93 (on the left) and by NHANESIII equations (on the right)

**Fig. 4:**
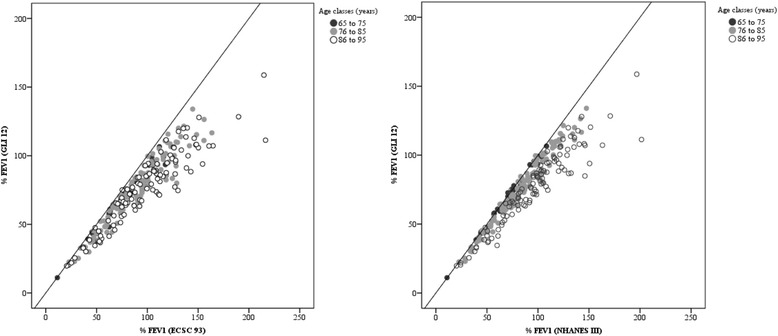
Scatterplot associating the results as a percentage of the predicted value for FEV_1_ from GLI12 reference equations with those obtained by ECSC93 (on the left) and by NHANESIII equations (on the right).

Using the Bland-Altman plot, the %FVC obtained from the ECSC equation may range from 5% below to 49% above the values derived from the GLI equation, and ranged from 12% below to 29% above those derived from the NHANES III equation. When %FEV_1_ is evaluated, it is possible to note that ECSC and NHANES III measurements may differ from GLI measurements by 9% below to 51% above the value and 11% below to 42% above the value, respectively (Figs. 1 and 2). Additionally, according to Figs. 3 and 4, it is possible to conclude that the dispersion increases with higher percentages, and the distance from the y = x line is more evident, particularly in older age classes. It seems that the GLI, NHANES III, and ECSC equations showed better agreement at younger ages.

### Lower limit of normality for spirometry parameters

The mean LLN value was also explored for FVC, FEV_1_ and %FEV_1_/FVC values calculated from each reference equation and within each sex. As can be seen, the ECSC equations provide the lowest mean LLN values for both FVC and FEV_1_ and the highest mean LLN value for %FEV_1_/FVC. Comparing these results with those from the GLI equations, the differences are 0.40 L, 0.40 L, and 10.4% for FVC, FEV_1_, and %FEV_1_/FVC, respectively (Table 2).

**Table 2:** LLN using NHANESIII, ECSC93, and GLI12 reference equations

			NHANESIII	ECSC93	GLI12
	n	Absolute values	Mean (SD)	Mean (SD)	Mean (SD)
Total					
FVC (l)	260	1.9 (0.71)	1.6 (0.56)	1.3 (0.61)	1.7 (0.46)
FEV_1_ (l)	260	1.4 (0.54)	1.1 (0.43)	0.9 (0.46)	1.3 (0.32)
FEV_1_/FVC (%)	260	70.9 (10.47)	62.7 (1.57)	71.9 (7.47)	61.5 (1.75)
Female					
FVC (l)	181	1.7 (0.51)	1.3 (0.36)	0.95 (0.33)	1.5 (0.20)
FEV_1_ (l)	181	1.2 (0.42)	0.9 (0.28)	0.7 (0.30)	1.1 (0.16)
FEV_1_/FVC (%)	181	72.03 (9.75)	63.3 (1.24)	76.9 (0.11)	62.0 (1.40)
Male					
FVC (l)	79	2.5 (0.80)	2.1 (0.57)	2.0 (0.49)	2.3 (0.35)
FEV_1_ (l)	79	1.7 (0.64)	1.5 (0.46)	1.4 (0.41)	1.7 (0.28)
FEV_1_/FVC (%)	79	68.3 (11.63)	61.5 (1.57)	60.7 (1.36)	60.3 (1.88)

### Spirometry interpretation

The impact of the application of a different set of reference equations on spirometry interpretation was also examined. Table 3 shows that the highest rate of obstruction was obtained using ECSC eqs. (52.3%) and the lowest using GLI eqs. (8.1%). These differences were more pronounced within the group of women where the prevalence of airway obstruction was 58.6% higher using the ECSC equation rather than the GLI equation. When using the GLI equation, a spirometric restrictive pattern was found in 29.6% of elderly subjects; the rate increased when men alone were studied (36.7%). The LLN for the FVC obtained using the ECSC equation showed the smallest rate for the restrictive pattern. NHANES III data, in general, revealed good agreement for the identification of both restrictive and obstructive disturbances. When the ECSC equations were used, both restrictive and obstructive disturbance proportions showed slight-to-fair agreement in women. In elderly men, the level of agreement increased when ECSC equations were used (Table 4).

**Table 3:** Obstructive and spirometric restrictive pattern disturbance proportions according to different interpretation methods

	Airway obstruction			Spirometric restrictive pattern		
	Total (*n* = 260)	Female (*n* = 181)	Male (*n* = 79)	Total (*n* =260)	Female (*n* = 181)	Male (*n* = 79)
NHANES III	39 (15.0%)	24 (13.3%)	15 (19.0%)	67 (25.8%)	44 (24.31%)	23 (29.1%)
ECSC93	136 (52.3%)	121 (66.9%)	15 (19.0%)	28 (10.8%)	11 (6.1%)	17 (21.5%)
GLI12	21 (8.1%)	15 (8.3%)	6 (7.59%)	77 (29.6%)	48 (25.52%)	29 (36.7%)

**Table 4:** Level of agreement for obstructive and spirometric restrictive pattern using GLI12 as a reference

GLI12		% Airway obstruction		% Spirometric restrictive pattern	
	Age (years)	Female (*n* = 181)	Male (*n* = 79)	Female (*n* = 181)	Male (*n* = 79)
NHANES III	All	0.74^c^	0.52^b^	0.77^c^	0.83^c^
	65–75	0.77	1	0.88	1
	76–85	0.78	0.3	0.83	0.86
	86–95	0.69	0.37	0.57	0.65
ECSC93	All	0.09^a^	0.52^b^	0.3^a^	0.64^c^
	65–75	0.21	1	0.39	0.75
	76–85	0.11	0.25	0.26	0.54
	86–95	0.05	0.44	0.29	0.65

(Kappa statistic; ^a^ - 0–0.4: slight to fair; ^b^ - 0.41 – 0.60: moderate; ^c^ - 0.61–0.99: good to very good)

## Discussion

In this study we assessed differences between values obtained from distinct sets of reference equations (ECSC, NHANES III, and GLI) in a sample of elderly subjects recruited at ECC. Moreover, the LLN was used for spirometry interpretation to discuss possible disparities. In fact, some differences were expected, as each reference equation was obtained by applying different statistical models to distinct populations.

Our findings indicate that higher values are calculated for %FVC and %FEV_1_ when the ECSC reference equations are used, allowing us to conclude that these equations provide lower predicted values. Higher predicted values for FVC and FEV_1_ are obtained with GLI equations, as our results show lower %FVC and %FEV_1_ values using these reference equations. However, %FEV_1_/FVC is underestimated using ECSC equations and overestimated using NHANES III equations. As LLN is obtained by considering predicted values, the highest LLNs are found for FVC and FEV_1_ using GLI equations; for FEV_1_/FVC, the highest LLNs are found using ECSC equations. These results suggest different rates of ventilatory disturbances based on spirometry. Since the LLN for FEV_1_/FVC differs by 10% between the GLI and ECSC equations and by 9% between the NHANES III and ECSC equations, the impact on the rate of airway obstructions was substancial when changing from ECSC equations. In our sample, ECSC reference equations identified more subjects with airway obstruction (52.3%) than either the NHANES III or ECSC reference equations. The highest LLN for FVC establishes a stricter criterion for a spirometric restrictive pattern. The GLI equation results in a higher mean LLN for FVC (100 mL and 400 mL compared with NHANES III and ECSC, respectively), and for that reason, the percentage of elderly subjects with restrictive defects was substancial (29.6%). The level of agreement was poor-to-slight between ventilatory defect proportions using the GLI equations as a reference compared with LLN criteria from ECSC equations and tended to be lower in more elderly subjects. Another relevant finding was that all differences observed were more evident in women and in subjects who were more elderly.

Previous studies were conducted to explore the applicability of GLI equations to different populations. Overall, the study populations were heterogeneous and comparisons with current data may be compromised. However, some considerations can be made.

We found discrepancies in airway obstruction and the spirometric restrictive pattern rate in most previous studies. Our data reflect more respiratory disturbances based on spirometry than in any other study. This finding is not surprising, as it is well known that lung volume decline is age-dependent [21–23]. Moreover, our sample included institutionalized elderly subjects; these individuals are potentially more dependent and have chronic diseases such as respiratory diseases. The prevalence of airway obstruction was markedly higher in only one study [12]. The authors calculated the predicted values for spirometry parameters using the same reference equations used in the present study. However, despite the young sample age, the data were from patients referred to pulmonary laboratories of tertiary hospitals, so it is probable that the prevalence of respiratory disease was higher.

Previous evidence showed both similarities and discrepancies when comparing respiratory disturbance rates between the three sets of reference equations. We found more airway obstructions using ECSC equations while more spirometric restrictive patterns were observed using GLI equations. Globally, previous studies have found that adopting the GLI reference equations had a minor impact on the rate of airway obstruction rather than on the rate of spirometric restrictive patterns where the impact was higher [12, 13, 17]. A French study [24] determined that GLI equations increased the rate of airway obstruction by 2.2% and the rate of spirometric restrictive patterns by 5.8% compared with ECSC equations. Between GLI and NHANES III equations, NHANES III equations identified 3% more airway obstruction in male subjects and 2.3% more obstruction in female subjects [25]. The primary differences between the referenced studies and ours are the age ranges and the older mean age, that was higher in the present study.

The lowest LLN for FVC and FEV_1_ using ECSC equations was found in other studies [17], although our study showed a higher FEV_1_/FVC ratio using ECSC equations, which contradicts previous studies [12, 17]. To justify this finding, we suspect that decreasing FVC and FEV_1_ values do not follow the same proportions, so the ratio does not show the same trend.

More recent studies were conducted to test the applicability of the GLI reference equations in comparison with those commonly used in the respective study populations [25–28]. A group of 1000 healthy, non-smoking, native Finnish subjects aged 18–83 years were evaluated and it was found that GLI equations underestimated lung volumes, and especially the FVC [26], which was a finding that contradicted our data. In this study, the authors compared the GLI reference equations with their own equations, and despite the lack of comparative studies, it has been suggested that Northern populations may have slightly larger lung volumes, a fact that can justify this disparity [26]. The mean %FEV_1_ predicted values derived from ECSC equations showed the lowest values in both female and male subjects in a Netherlands study [27], which are findings that contradict ours. An important difference between the two studies is that the mean age was much lower in the previous study than in ours. However, in a sample of elderly Brazilian subjects with a mean age approximately equal to that in our study, the LLN was similar to that in our study when using GLI equations [28]. Linares-Perdomo et al. [25] found that differences between NHANES III and GLI equations are generally small and probably not clinically important for most patients. However, the authors suggest caution when selecting prediction equations for elderly patients, and especially for tall and short patients. In fact, our sample is characterized by a significant percentage of elderly women patients (69.6%) with a mean height of 1.56 m.

The ability of elderly individuals to achieve a good spirometry performance deserves further discussion. To our knowledge, the first study concerning the quality control of spirometry performance in elderly individuals was performed in 2000. The primary results showed that the sources of variability in performance are numerous, and include motor and sensory deficits, dementia, depression, and malnutrition [29]. Despite the fact that more time was required to complete the examination to obtain the necessary quality standards, in this study, all spirometry tests adhered to international guidelines for acceptability [1] and repeatability [18]. According to Pezzoli et al. [30], age cannot be considered a risk factor for a poor spirometry performance, as in their study the majority of elderly patients with no cognitive or functional impairment underwent spirometry tests according to international guidelines.

The study subjects ranged in age from 65 to 95 years. ECSC reference equations were obtained from a sample of subjects of European descent with ages that ranged from 18 to 70 years. In all, 96.2% of the elderly subjects included in our study were more than 70 years old. NHANES III included a random sample of the US population with an age range of 8–80 years living in households. It provides regression equations for predicted values and LLNs for the three primary racial/ethnic groups in that country: Caucasians, African-Americans, and Mexican-Americans. The applicability of NHANES III data to the present sample is associated with two major limitations. First, 76.2% of the elderly subjects in the previous study were more than 80 years old, and second, the U.S. population has different anthropometric data characteristics than those found in Europe. The predicted values for subjects older than the maximum ages evaluated by the NHANES III and ECSC were extrapolated beyond 80 and 70 years, respectively. This fact may have caused bias in the results; however, these reference equations are still used in some LFLs and in community-based spirometry studies, so it is necessary to test their applicability to elderly subjects.

The spirometric restrictive pattern should be interpreted carefully. In fact, the measurement of FVC by spirometry is useful to exclude restriction, but there are limitations to the identification of disturbances [31, 32]. An accurate restrictive pattern can only be identified by measuring the total lung capacity (TLC).

An ageing population represents the reality in most developed countries, and in recent years, a set of initiatives has been developed for active ageing that includes more opportunities for health and social participation to enhance the quality of life in the elderly population. Ageing is also accompanied by a decline in lung function, and this population is more susceptible to acute and chronic pulmonary diseases. We believe that the main conclusions of this study determined two goals for further investigations. One is the requirement to continue the validation of GLI equations in elderly populations, assessing discrepancies in the results between men and women. The GLI’s new update is the first approach that allows calculations of the predicted, lower, and upper limits of normality for subjects up to 95 years old. Another advantage of the GLI equations is that z-scores can be calculated, allowing clinicians to interpret lung function results independent of age, height, sex, and ethnic group, which decreases the possible bias of using predicted values. Therefore, testing the benefit of using z-scores in an elderly population will update lung function procedures, as this practice is only used in paediatric populations.

## Conclusions

The purpose of this study was not to conclude which reference equation should be used for elderly subjects but rather to demonstrate the impact of each reference equation on the percentages of predicted values and proportions of ventilatory disturbances. Moreover, it is important to show how the clinical intervention and patient’s follow up may be compromised by the reference equation used. In fact, it is possible to conclude from this study that changing from ECSC to GLI equations will have a meaningful impact on classifications with fewer obstructed and more restricted subjects. The data in the present study showed meaningful differences in spirometric outcomes, and consequently, in the proportion of ventilatory disturbances.

## Abbreviations

ATS: American Thoracic Society
CNPD: Portuguese Data Protection Authority
ECC: Elder care centres
ECSC: European Community of Steel and Coal
ERS: European Respiratory Society
FEV_1_: Forced expiratory volume in 1 s
FVC: Forced vital capacity
GERIA: Geriatric Study in Portugal on Health Effects of Air Quality
GLI: Global Lung Initiative
LLN: Lower limit of normality
NHANES III: National Health and Nutrition Assessment Survey
SD: Standard deviation
TLC: Total lung capacity
